# Predictors of subclinical atherosclerosis evaluated by carotid intima-media thickness in asymptomatic young women with type 1 diabetes mellitus

**DOI:** 10.1590/2359-3997000000255

**Published:** 2017-02-01

**Authors:** Rosane Kupfer, Manuella Rangel Larrúbia, Isabela Bussade, Joana Rodrigues Dantas Pereira, Giovanna A. Balarini Lima, Marcio Antonio Epifanio, Claudio Domenico Sahione Schettino, Denise Prado Momesso

**Affiliations:** 1 Departamento de Diabetes Instituto Estadual de Diabetes e Endocrinologia Luiz Capriglione Pontifícia Universidade Católica do Rio de Janeiro Rio de Janeiro RJ Brasil Departamento de Diabetes, Instituto Estadual de Diabetes e Endocrinologia Luiz Capriglione (IEDE), Pontifícia Universidade Católica do Rio de Janeiro (PUC-RJ), Rio de Janeiro, RJ, Brasil; 2 Gaveacor Rio de Janeiro RJ Brasil Gaveacor – Diagnóstico Cardiológico, Rio de Janeiro, RJ, Brasil

**Keywords:** Type 1 diabetes, central obesity, subclinical atherosclerosis, carotid intima-media thickness, insulin resistance

## Abstract

**Objective:**

This study aimed to evaluate the occurrence and clinical predictors of subclinical atherosclerosis in asymptomatic, young adult women with type 1 DM.

**Subjects and methods:**

The study included 45 women with type 1 diabetes mellitus (DM) (aged 36 ± 9 years) who underwent carotid Doppler ultrasound evaluation to determine the carotid artery intima-media thickness (CIMT) and to assess the occurrence of carotid artery plaques. Insulin sensitivity was assessed by estimated glucose disposal rate (eGDR), and metabolic syndrome (MS) was defined by the World Health Organization criteria.

**Results:**

The cohort had a mean age of 36 ± 9 years, diabetes duration of 18.1 ± 9.5 years, and body mass index (BMI) of 24.6 ± 2.4 kg/m^2^. MS was present in 44.4% of the participants. The CIMT was 0.25 ± 0.28 mm, and the prevalence of carotid artery plaques was 13%. CIMT correlated positively with hypertension (p = 0.04) and waist-to-hip ratio (r = 0.37, p = 0.012). The presence of carotid artery plaques correlated positively with age (p = 0.018) and hypertension (p = 0.017). eGDR correlated negatively with CIMT (r = -0.39, p = 0.009) and carotid plaques (p = 0.04). Albuminuria showed a correlation trend with CIMT (p = 0.06). Patients with carotid artery plaques were older, had a higher prevalence of hypertension, and lower eGDR. No correlation was found between CIMT and carotid plaques with diabetes duration, MS, BMI, cholesterol profile, glycated hemoglobin, high-sensitivity C-reactive protein, or fibrinogen.

**Conclusion:**

Insulin resistance, central obesity, hypertension, and older age were predictors of subclinical atherosclerosis in asymptomatic, young adult women with type 1 DM.

## INTRODUCTION

Type 1 diabetes mellitus (DM) is associated with a high incidence of atherosclerotic disease and early mortality. The process of vascular changes starts at a young age in patients with type 1 DM, even though the atherosclerotic disease may only manifest clinically later in life (1-4). Chronic hyperglycemia may predispose to inflammation, endothelial dysfunction, and changes in coagulation, which in turn are associated with cardiovascular diseases (CVD). In addition, more youths today have overweight and central obesity, both predisposing to insulin resistance and hypertension and further increasing the risk of CVD in individuals with type 1 DM (5-8). Based on that, the identification of clinical predictors of subclinical atherosclerosis is essential to prevent the morbidity and mortality associated with CVD in these patients.

Subclinical atherosclerosis may be assessed by noninvasive techniques such as carotid artery ultrasound, which measures the carotid intima-media thickness (CIMT) and evaluates the occurrence of carotid artery plaques. Increased CIMT is a well-established surrogate for subclinical atherosclerosis and a risk factor for myocardial infarction and stroke. Carotid plaques have also been shown to be a useful predictor of coronary atherosclerosis (9-13).

Based on the above, the aims of this study were to evaluate the occurrence and clinical predictors of subclinical atherosclerosis in asymptomatic, young adult women with type 1 DM. For this purpose, subclinical atherosclerosis was evaluated by carotid Doppler ultrasound with measurement of CIMT and assessment of the presence of carotid artery plaques.

## SUBJECTS AND METHODS

This was a cross-sectional, uncontrolled study evaluating women with type 1 DM consecutively attending the outpatient diabetes clinic of the *Instituto Estadual de Diabetes e Endocrinologia Luiz Capriglione* (IEDE) over a 6-month period. A total of 45 women were enrolled. The inclusion criteria were a diagnosis of type 1 DM with a disease duration longer than 10 years, regular insulin therapy with multiple injections, positive anti-glutamic acid decarboxylase (GAD) autoantibodies, regular menstrual periods, and age older than 18 years. The exclusion criteria were a past history of CVD (myocardial infarction, angina, stroke and/or events in other major vessels), symptoms of CVD, and ovarian failure.

Weight (in kilograms [kg]) and height (in meters [m]) were measured with the subjects wearing only undergarments. Body mass index (BMI) was calculated as the body weight divided by the squared height (kg/m^2^). With the subjects standing with their heels together, we measured the waist circumference (WC) midway between the lowest rib margin and the iliac crest, and the hip circumference at the widest circumference over the greater trochanters, both in centimeters (cm). After obtaining both results, we calculated the waist-to-hip ratio (WHR).

Fasting blood samples were collected and analyzed for measurement of glycated hemoglobin (HbA1c) and lipids. HbA1c concentrations were measured by high- pressure liquid chromatography (Variant II, Biorad), using a reference range of 4% to 6%. Serum total cholesterol, HDL cholesterol, and triglycerides were measured with an enzymatic colorimetric assay (Advia, Siemens). LDL cholesterol was calculated using the Friedewald formula. Albuminuria (urinary albumin excretion rate) was determined in a 24-hour urine sample by the nephelometric method (BNII, Siemens). High-sensitivity C-reactive protein (hs-CRP) and fibrinogen were measured by immunoturbidimetric and nephelometric assays, respectively (both by Advia, Siemens).

The insulin sensitivity was calculated using the estimated glucose disposal rate (eGDR), previously validated by Williams and cols. (14), according to the following equation: 24.31- (12.22 x WHR) – 3.29 x HT) – 0.57 x HbA1c (in mg.kg^-1^.min^-1^), in which HT represents hypertension (14). An elevated eGDR value represents a high insulin sensitivity (14). The daily insulin dosage was calculated at baseline in units per kilogram of body weight.

Metabolic syndrome (MS) was defined according to the modified World Health Organization (WHO) consensus criteria (15). To establish the diagnosis of MS, the modified WHO criteria requires the presence of glucose intolerance or diabetes and/or insulin resistance plus two of the following: 1) hypertension, defined as the use of antihypertensive treatment and/or elevated blood pressure (systolic ≥ 160 mmHg or ≥ diastolic 90 mmHg); 2) dyslipidemia, defined as an elevated plasma triglyceride level (≥ 150 mg/dL) and/or a low HDL cholesterol level (< 39 mg/dL in women); 3) obesity, defined as a high WHR (≥ 0.85 in women); 4) albuminuria (urinary albumin excretion rate ≥ 20 µg/min).

Each patient underwent a complete Doppler echocardiographic evaluation of both common carotid arteries according to the American Society of Echocardiography guidelines (16). The echocardiographic evaluations were performed with an M3S transducer in a VIVID 7 (GE, USA) equipment following standard techniques. Doppler echocardiography studies were performed by the same cardiologist with a high experience in echocardiography and a high interstudy reproducibility, who was unaware of the clinical data. All measurements were performed during the examination using longitudinal images obtained by the automatic calibrator of the equipment. The evaluations were conducted with the subjects in the supine position with their heads elevated at 15^o^ and turned to the opposite side of the transducer. The images were recorded into a computerized database. CIMT was defined as the distance between the media-adventitia interface and the lumen-intima interface. We measured CIMT twice on each side (right and left) and a mean value of both these measurements was obtained for each side, with the highest value (right or left) considered for the analysis. The common carotid arteries were evaluated at a distance of 1 to 3 cm from their bifurcation, and the internal carotid arteries were evaluated at their initial 2 cm. Carotid artery plaques were evaluated and reported.

### Statistical analysis

The statistical analysis was performed using the software SPSS 17.0 (SPSS Inc., Chicago, IL, USA) and the significance threshold was set at 0.05. Data are presented as mean ± standard deviation (SD) for continuous variables and as absolute numbers (relative frequencies) for discrete variables. The Mann-Whitney test and the chi-square test were used for comparisons between groups. Spearman correlation coefficient was calculated where indicated.

The study was approved by the Ethics Committee of the *Instituto Estadual de Diabetes e Endocrinologia Luiz Capriglione*. All patients agreed to participate in the research and signed a written informed consent form.

## RESULTS

The clinical characteristics of the 45 women with type 1 DM enrolled in the study are shown in Table 1. The study evaluated young adult women (mean age = 36.2 ± 9.5 years) with type 1 DM of long duration (mean duration 18.1 ± 9.5 years). The prevalence of MS according to the WHO criteria was 44.4%, and none of the patients were obese (mean BMI 24.6 ± 2.4 kg/m^2^). The mean CIMT was 0.25 ± 0.28 mm, and the prevalence of carotid plaques was 13% (n = 6).

**Table 1:** Baseline characteristics of 45 young adult women with type 1 diabetes

Age (years)	36.2 ± 9.5
Diabetes duration (years)	18.1 ± 9.5
Presence of metabolic syndrome	20 (44.4%)
Presence of hypertension	16 (35.6%)
Cigarette smoking	4 (8.9%)
BMI (kg/m^2^)	24.6 ± 2.4
WC (cm)	83.9 ± 11.51
WHR	0.86 ± 0.07
LDL cholesterol (mg/dL)	99.8 ± 33.4
HDL cholesterol (mg/dL)	53.82 ± 13.8
Triglycerides (mg/dL)	99.88 ± 71.63
Insulin dosage (U/kg/dia)	0.79 ± 0.24
HbA1c (%)	8.5 ± 2.0
CIMT (mm)	0.25 ± 0.28
Carotid artery plaques	6 (13%)

BMI: body mass index; WC: waist circumference; WHR: waist-to-hip ratio; HbA1c: glycated hemoglobin; CIMT: carotid intima-media thickness. The values are expressed as mean ± standard deviation, or number of patients and percentage.

Table 2 shows the results of univariate analysis of risk factors associated with CIMT and with the presence of carotid artery plaques. CIMT correlated positively with hypertension (p = 0.04) and WHR (r = 0.37, p = 0.012). The presence of carotid artery plaques, determined by ultrasound, correlated positively with age (p = 0.018) and hypertension (p = 0.017). Insulin sensitivity, assessed with the eGDR, correlated negatively with CIMT and carotid plaques (p = 0.009 and p = 0.04, respectively). The albuminuria level showed a correlation trend with the presence of carotid artery plaques (r = -0.39, p = 0.06). No correlation was found between CIMT and diabetes duration, MS, BMI, WC, cholesterol profile, HbA1c, hs-CRP, or fibrinogen.

**Table 2:** Univariate analysis of risk factors associated with subclinical carotid atherosclerosis measured by carotid intima-media thickness and the presence of carotid artery plaques

	Carotid intima-media thickness	Carotid artery plaques
Age (years)	r = 0.23 p = 0.13	p = 0.018*
Diabetes duration (years)	r = 0.11 p = 0.13	p = 0.30
Presence of metabolic syndrome	p = 0.32	p = 0.38
Presence of hypertension	p = 0.04*	p = 0.017*
Cigarette smoking	p = 0.82	p = 0.45
BMI (kg/m^2^)	r = 0.01 p = 0.94	p = 0.27
WC (cm)	r = 0.24 p = 0.12	p = 0.46
WHR	r = 0.37 p = 0.012*	p = 0.68
LDL cholesterol (mg/dL)	r = - 0.004 p = 0.98	p = 0.11
HDL cholesterol (mg/dL)	r = 0.03 p = 0.85	p = 0.44
Triglycerides (mg/dL)	r = -0.008 p = 0.96	p = 0.31
Albuminuria (µg/min)	r = 0.096 p = 0.53	p = 0.06
HbA1c (%)	r = 0.13 p = 0.41	p = 0.86
hs-CRP	r = 0.04 p = 0.82	p = 0.77
Fibrinogen	r = - 0.16 p = 0.37	p = 0.98
eGDR	r = - 0.39 p = 0.009*	p = 0.04*

BMI: body mass index; WC: waist circumference; WHR: waist-to-hip ratio; HbA1c: glycated hemoglobin; hs-CRP: high-sensitivity C-reactive protein; eGDR: estimated glucose disposal rate. * Statistical significance (p < 0.05).

Carotid artery plaques were observed in six patients (13%). Table 3 shows the clinical characteristics of the patients with and without such plaques. Women with type 1 DM with carotid artery plaques were older, had a higher prevalence of hypertension, and lower eGDR, reflecting higher insulin resistance. Patients with carotid plaques showed a trend toward higher albuminuria levels compared with patients without plaques (43.9 ± 53.64 µg/min and 10.08 ± 9.02 µg/min, respectively, p = 0.06).

**Table 3:** Clinical characteristics of asymptomatic women with type 1 DM with and without carotid artery plaques

	With carotid artery plaques (n = 6)	Without carotid artery plaques (n = 39)	P value
Age (years)	44.3 ± 6.9	34.9 ± 9.3	0.018*
Diabetes duration (years)	22.3 ± 11.2	17.5 ± 9.3	0.30
Presence of metabolic syndrome	4 (66.7%)	16 (41.0%)	0.38
Presence of hypertension	5 (84%)	11 (28%)	0.017*
Cigarette smoking	1 (16.7%)	3 (7.7%)	0.45
BMI (kg/m^2^)	26.04 ± 2.3	24.43 ± 4.6	0.29
WC (cm)	85 ± 41	83 ± 66	0.60
WHR	1.0 ± 0.88	1.08 ± 0.85	0.46
LDL cholesterol (mg/dL)	116.8 ± 36.9	97.12 ± 32.6	0.10
HDL cholesterol (mg/dL)	49.2 ± 11.3	54.5 ± 13.9	0.44
HbA1c (%)	8.7 ± 2.6	8.6 ± 1.9	0.87
Albuminuria (µg/min)	26.5 (7.8-75.5)	7.0 (5.0-13.10)	0.06
hs-CRP	2.06 ± 1.97	4.67 ± 1.04	0.46
eGDR	4.98 ± 2.0	7.16 ± 2.47	0.04*

BMI: body mass index; WC: waist circumference; WHR: waist-to-hip ratio; HbA1c: glycated hemoglobin; hs-CRP: high-sensitivity C-reactive protein; eGDR: estimated glucose disposal rate. Values are expressed as mean ± standard deviation, median (interquartile range), or number of patients and percentage. * Statistical significance (p < 0.05).

## DISCUSSION

The occurrence of CVD is an important cause of morbidity and mortality in type 1 DM (1-4). Therefore, it is imperative to identify factors predictive of subclinical atherosclerosis to diagnose early, treat, and prevent the occurrence of CVD. For that purpose, we evaluated asymptomatic, young adult women with type 1 DM with no past history of CVD. We targeted young women with type 1 DM in our study since this group experiences a greater relative risk of presenting CVD when compared with men and individuals without DM (2,3). We included in this study patients with DM of long duration (18.1 ± 9.5 years), in order to best evaluate the effects of type 1 DM in the atherosclerotic process. We included only non-menopausal women, since the incidence of CVD increases after menopause (17).

In the present study, subclinical atherosclerosis was evaluated by carotid artery ultrasound and defined as the occurrence of an increased CIMT and/or presence of carotid artery plaques. In our cohort of young adult women with type 1 DM, we found a 13% prevalence of carotid artery plaques, consistent with emerging subclinical atherosclerotic disease, and a mean CIMT of 0.25 ± 0.28 mm. A study in 150 patients with long-standing type 1 DM has found a lower prevalence of subclinical atherosclerosis (18). In contrast, other studies have reported increased CIMT in children and adolescents with type 1 DM, demonstrating that the atherosclerotic process may start at a young age in these patients (19,20). Our results provide further evidence of the importance of screening for subclinical CVD in women with type 1 DM.

Central obesity, evaluated with the WHR, correlated positively with CIMT. The WHR is a useful anthropometric measurement of central fat deposition and overcomes the limitations of the WC (21). Indeed, we found correlations between WC and CIMT and carotid plaques. Furthermore, none of the patients evaluated in our study were obese, and BMI was not associated with CIMT or carotid plaques. These results suggest that the pattern of central fat deposition in type 1 DM may also be associated with increased risk of atherosclerotic CVD and negative metabolic effects, similar to the findings described in type 2 DM patients (8,22-24).

We also observed that insulin resistance was associated with subclinical predictors of atherosclerosis. In type 1 DM, it is often difficult to accurately identify insulin resistance with clinical parameters alone. The eGDR has been validated as an easy method to evaluate insulin resistance in type 1 DM, with higher values indicating increased insulin sensitivity (14). In the present study, eGDR correlated negatively with CIMT and with carotid plaques. In fact, it has been demonstrated that insulin resistance is a predictor of coronary artery disease in patients with type 1 DM (25-28). To the best of our knowledge, this is the first study describing the evaluation of insulin resistance by eGDR as a surrogate marker of subclinical atherosclerosis in type 1 DM.

Central obesity and insulin resistance are important clinical features of the MS (29,30). However, we found no association between MS and subclinical atherosclerosis measured by carotid artery ultrasound, thus indicating that the presence of MS defined by the WHO criteria did not add a predictive value for subclinical atherosclerosis detection in our population. The MS has been shown to predict major complication outcomes in type 1 DM in several clinical studies, but in other studies, the presence of MS did not add a significant predictive prognostic value to conventional CVD risk factors alone (25,28,31-34). We used the WHO criteria for the MS classification since it has exhibited the highest sensitivity to discriminate negative outcomes in patients with type 1 DM in prior studies (28,31). Indeed, a previous study from our group has critically analyzed different criteria for MS in type 1 DM patients and observed that the WHO criteria were the preferred method to identify MS in this population when compared with to the IDF and NCEP-ATP III criteria (31).

Hypertension is a well-established, traditional CVD risk factor. Clinical trials have shown that diastolic blood pressure in the highest quintile, even without the added risks of high cholesterol and smoking, still contributes significantly to the risk of atherosclerosis (35,36). Our data corroborate this knowledge, as hypertension correlated positively with CIMT and carotid plaques and was more frequently diagnosed in patients with carotid obstruction. It is important to notice that despite the fact that all hypertensive women in this study had blood pressure levels controlled with medication, an effect of hypertension on the atherosclerotic process was still observed.

Increased age was associated with carotid artery plaques and obstruction but not with CIMT. There are controversies in the literature regarding the association of age and CIMT, in which positive (20,36) and negative (19) results have been reported, but none of these studies have evaluated the presence of carotid plaques.

Albuminuria has been recently recognized as a risk factor for CVD, both in patients with DM as well as in those with hypertension, and is considered an initial marker of diabetic nephropathy (37,38). We observed a trend of an association between albuminuria levels and the presence of carotid artery plaques, with patients with carotid obstruction displaying increased levels of albuminuria, as also described by other authors (37,38).

We observed no association between DM duration and subclinical atherosclerosis. This finding contrasts with previous studies that have demonstrated that CIMT increases with the duration of type 1 DM (19,20,36). In these studies, the duration of the disease was shorter than that in our study, which might explain our findings. Furthermore, we found no association between CIMT and glycemic control measured by HbA1c, as reported in other studies (19,20). Our results are similar to those of another study in a cohort of 603 patients with type 1 DM followed up for 10 years, which found that a better glycemic control was unable to predict the occurrence of coronary artery disease (CAD), in contrast to insulin resistance measured by eGDR, which emerged as a predictor of CAD endpoints (27). Furthermore, since this was a cross-sectional study, we had no information regarding the glycemic control of the patients before the study, which could have had an impact on these findings. Finally, the follow-up duration probably needs to be longer to confirm an association between hyperglycemia and CVD. In fact, in the Diabetes Control and Complication Trial/Epidemiology of Diabetes Interventions and Complication (DCCT/EDIC) study, a longer observation time was needed to confirm the association between hyperglycemia and CVD, especially in a population of younger patients (39).

Laboratory inflammation markers have been used as predictive tools for atherosclerosis in patients with type 2 DM (36,40-43). In our study, we found no association between the inflammation markers hs-PCR and fibrinogen with subclinical atherosclerosis in patients with type 1 DM. A study in children and adolescents with type 1 DM has shown that levels of plasminogen activator factor-1 (PAI-1) correlated positively with CIMT (42). Nitric oxide, an oxidative stress marker, also correlated with CIMT in young patients with type 1 DM, suggesting a possible role of oxidative stress in the development of atherosclerosis (20). Data from the DCCT/EDIC study have shown in patients with type 1 DM that plasma homocysteine correlates with CIMT but not with CIMT progression; based on this finding, routine homocysteine measurement in patients with type 1 DM found no support (43). Thus, further studies are needed to evaluate the clinical impact of laboratory inflammation markers in the prediction of subclinical atherosclerosis in type 1 DM.

The present study has several limitations. First, due to its cross-sectional design, our findings could not prove causal relationships between risk factors and subclinical atherosclerosis. Additionally, we evaluated only young, non-menopausal women, so our results must be interpreted considering that they may be not valid for male patients with type 1 DM. Since we did not include a control group of patients without diabetes, the most likely bias in this context would be the fact that other factors, apart from the DM, could have been responsible for the carotid abnormalities that we found. This is a pilot study, and additional studies with larger cohorts with increased prevalence of subclinical atherosclerosis and longitudinal trials are still needed to elucidate further the clinical risk factors for subclinical atherosclerosis diagnosed by carotid artery ultrasonography.

In conclusion, the present study provided important evidence that central obesity, insulin resistance, hypertension, and older age were clinical predictors of subclinical atherosclerosis in asymptomatic, nonobese, young adult women with type 1 DM. Subclinical atherosclerosis was assessed by CIMT measurement and by the presence of carotid plaques, which are well-established markers of CVD. The clinical evaluation of these predictors of subclinical atherosclerosis might, therefore, guide CVD risk stratification and prevention of type 1 DM.
